# Letter from Mr. Assalini to Mr. Want

**Published:** 1814-12

**Authors:** Paul Assalini

**Affiliations:** 7, Manchester-street


*For the Medical and Physical Journal.*

*Letter from* Mr. ASSALINI *to* Mr. WANT.

*My Dear Sir,*

I YESTERDAY found, in the Number of your excellent Journal for November, an article which concerns me, and reflects on the surgeons of Italy. I never suffer criticism to give me a moment's uneasiness. I am equally indifferent to praise or censure. Time will decide in any points of difference between us.

I hope by this time you have read my *Manuale di Chirurgia.* You will discover in that publication, as in the works of Nannoni, Flajani, Molinelli, Manzoni, Ruggieri, Paletta,

Monteggia,

Monteggia, and other Italian authors, that the writer, in drawing up that notice, would have been more prudent in first learning what they have said *against the propriety* of dividing the inner coat of the artery in the treatment of aneurisms, before he accuses them of inattention to the fact of its rupture on the application of the ligature.

We were fully aware of the observations of Dr. Jones; but his conclusions are by no means confirmed in a great many cases, of which the man who sunk from hæmorrhagy at Guy's Hospital is a melancholy proof.

Experiments recently made on the horse and dog, an account of which I communicated to you at our last interview, prove that the rupture of the inner coat of an artery is not sufficient for the obliteration of its cavity, or for the prevention of hæmorrhagy. The dog, whose carotid arteries were tied in the manner recommended by Jones, died of hæmorrhage. The carotid of the horse, on which a ligature was applied in the same manner, was found, on examination, to be still pervious; while the other carotid, on which *my compressor arteriæ* was applied, in the course of twenty-four hours was most completely obliterated. On opening it, two clots of firm coagulable lymph were found, one on each side of the stricture made by the ligature. You will oblige me by examining those preparations, and giving such account of them in your next Number as you may deem necessary.*

7, *Manchester-street*, PAUL ASSALINI.

*Nov.* 21, 1814.

*For*

---

FROM THE EDITORS.

* It has been explained, and we believe satisfactorily, to Mr. Assalini, that we are incapable of making any statement *as a reflection* upon the surgeons of Italy, and still less upon so celebrated and intelligent a practitioner as himself, of whose abilities we entertain the highest opinion. In making the remark respecting the rupture of the coat of an artery consequent to the use of the ligature, we were actuated by a desire, not so much to proclaim the ignorance of our continental friends, as to seize the opportunity of giving general circulation to a fact, which seemed to us of the utmost importance in our operations on the trunk of an aneurismal or wounded artery, and which we have reason to believe is yet unknown to many of our junior practitioners. It appears from the above statement of the professor, that we have been unjust in ascribing to the Italians an ignorance of Dr. Jones's experiments, because they were not only acquainted with them, but have disputed the accuracy of the doctor's conclusions. Though we believe believe the rupture of the inner coat of an artery to be essential to the speedy union of its sides, and consequently to the success of an operation upon it: we think it important to present our readers with the fact contained in this letter, that surgeons of the first rank in Italy are of a contrary opinion; and it is equally certain that some practitioners of eminence in this metropolis agree with them as to its being neither essential or useful. We find on inquiry that the experiment at St. George's was made under mistaken impressions; and, although most, if not all, of the gentlemen then present would have joined us in asserting the truth of our previous statement, it was afterwards distinctly ascertained to have been founded in misconception.

